# Herpesviruses infections associations in people with young onset dementia – Herpes simplex infection appears to be relevant

**DOI:** 10.1002/alz.093045

**Published:** 2025-01-03

**Authors:** Akram A. Hosseini, Peter A.C Maple, Radu Tanasescu, Bruno Gran

**Affiliations:** ^1^ Academic Neurology, Nottingham University Hospitals NHS Trust, Nottingham, Nottinghamshire United Kingdom; ^2^ Sir Peter Mansfield Imaging Centre, University of Nottingham, Nottingham United Kingdom; ^3^ Nottingham University Hospital Trust, Nottingham, Nottingham United Kingdom; ^4^ Nottingham University Hospitals NHS Trust, Nottingham United Kingdom

## Abstract

**Background:**

Early Onset Alzheimer’s Disease (EOAD) is thought to result from a combination of environmental, genetic, and lifestyle factors. Herpesvirus infections may contribute to the development of EOAD and the objective of our study is to identify potential associations between herpes virus infections and the risk of developing EOAD.

**Method:**

Amyloid‐status Alzheimer’s disease was diagnosed based on clinical history and ATN criteria, determined by the measuring the Amyloidß42:40 ratio, tau, and 181‐Phospho‐tau in the cerebrospinal fluid. EOAD was defined as the onset of symptoms before the age of 65. Antibodies to herpesviruses were detected in patients’ sera using commercially available enzyme immunoassays. The following markers were tested: cytomegalovirus IgG, total (HSV1/HSV2) herpes simplex virus IgG, IgM and IgA, and varicella‐zoster virus (VZV) IgG. The Mann‐Whitney U test and the Fisher’s exact test assessed differences in population and categorized data, with statistical significance set at p<0.05.

**Result:**

Sera from 20 individuals with Amyloid‐status EOAD and 20 age‐ and sex‐matched healthy controls (HCs) were tested. There were 13 females and seven males in the EOAD and HCs groups with a mean age of 60.4 years and 60.7 years, respectively. All individuals were VZV IgG seropositive with geometric mean levels of 967 mIU/ml and 827 mIU/ml in EOAD and HCs, respectively. The seroprevalences of CMV IgG were 45.0% and 35.0% in EOAD and HCs, respectively. The geometric mean CMV IgG levels were significantly higher (p = 0.029) in EOAD (678 Paul Ehrlich International units/ml) versus HCs (380 Paul Ehrlich International units/ml). The seroprevalences of HSV IgG were 90.0% in EOAD and 65.0% in HCs (difference not significant). Total HSV IgG levels were similar in EOAD (geometric mean level = 594 EU/ml) and HCs (geometric mean level = 640 EU/ml). Two individuals with EOAD and none in the HCs group showed evidence of HSV reactivation.

**Conclusion:**

The sample sets tested are small; however, testing is ongoing. Currently, it appears that individuals with EOAD have higher levels of CMV IgG compared to healthy controls.

